# Feasibility of non-intubated anesthesia and regional block for thoracoscopic surgery under spontaneous respiration: a prospective cohort study

**DOI:** 10.1590/1414-431X20198645

**Published:** 2019-12-20

**Authors:** Hanwei Li, Daiqiang Huang, Kun Qiao, Zheng Wang, Shiyuan Xu

**Affiliations:** 1 Department of Anesthesiology, Zhujiang Hospital of Southern Medical University, Guangzhou, Guangdong, China; 2 Department of Anesthesiology, Shenzhen People's Hospital, Shenzhen Anesthesiology Engineering Center, Shenzhen, Guangdong, China; 3 Department of Thoracic Surgery, Shenzhen Third People's Hospital, Guangdong Medical College, Shenzhen, Guangdong, China; 4 Department of Thoracic Surgery, Shenzhen People's Hospital, Shenzhen, Guangdong, China

**Keywords:** Non-intubated anesthesia, Paravertebral block, Postoperative recovery, Thoracoscopy, Spontaneous respiration

## Abstract

Data about the feasibility and safety of thoracoscopic surgery under non-intubated anesthesia and regional block are limited. In this prospective study, 57 consecutive patients scheduled for thoracoscopic surgery were enrolled. Patients were sedated with dexmedetomidine and anesthetized with propofol and remifentanil. Ropivacaine was used for intercostal nerve and paravertebral block. Lidocaine was used for vagal block. The primary outcomes were mean arterial pressure (MAP), heart rate (HR), oxygen saturation, and end-tidal carbon dioxide partial pressure (ETCO_2_) at T0 (pre-anesthesia), T1 (immediately after laryngeal mask/nasopharyngeal airway placement), T2 (immediately after skin incision), T3 (10 min after opening the chest), T4 (end of surgery), and T5 (immediately after laryngeal mask/nasopharyngeal airway removal). One patient required conversion to intubation, 15 developed intraoperative hypotension, and two had hypoxemia. MAP at T0 and T5 was higher than at T1–T4; MAP at T3 was lower (P<0.05 *vs* other time points). HR at T0 and T5 was higher (P<0.05 *vs* other time points). ETCO_2_ at T2 and T3 was higher (P<0.05 *vs* other time points). Arterial pH, PCO_2_, and lactic acid at T1 differed from values at T0 and T2 (P<0.05). The Quality of Recovery-15 (QoR-15) score at 24 h was lower (P<0.05). One patient experienced dysphoria during recovery. Thoracoscopic surgery with regional block under direct thoracoscopic vision is a feasible and safe alternative to conventional surgery under general anesthesia, intubation, and one-lung ventilation.

## Introduction

Thoracic surgery has evolved rapidly in recent decades due to the development and refinement of one-lung isolation procedures. Intubated surgery under general anesthesia with one-lung ventilation is now recognized as a routine methodology (1). Video-assisted thoracoscopic surgery (VATS) is a minimally invasive technique and is conventionally used in intubated patients under general anesthesia. During VATS, one-lung ventilation can be achieved using a bronchial blocker or a double-lumen tube. Because intubation and general anesthesia are not without risks, there has been increasing interest in the use of thoracic surgery without tracheal intubation to achieve a stable perioperative status and complete the treatment in patients at high risk of complications during intubated general anesthesia. Encouragingly, non-intubated thoracoscopic procedures are feasible and safe for multiple thoracic diseases such as resection of lung parenchyma for pulmonary tumors (2), biopsy of interstitial lung disease (3), bullectomy for pneumothorax (4), decortication for thoracic empyema (5), and excision of mediastinal tumors (6).

Conventional surgery with double-lumen intubation is not recommended for those who do not require lung isolation (7). Non-intubated thoracoscopic procedures are distinct from conventional intubated surgery under general anesthesia in that the patient is able to breathe spontaneously under regional anesthesia (2,3,8,9). Non-intubated thoracoscopic surgery avoids the need for tracheal intubation, lung isolation devices, neuromuscular blocking agents, and mechanical ventilation, thereby lowering the risks of complications such as lung infection, lung injury, cardiac dysfunction, and bronchospasm (10,11). Even biopsies for interstitial lung disease performed under intubated general anesthesia are associated with a mortality and morbidity of 5.8 and 14.7%, respectively (12,13), highlighting the need for the development of less invasive surgical procedures.

Nevertheless, there is a lack of studies regarding the use of non-intubated anesthesia for thoracic operations. Therefore, the present study was undertaken to assess the feasibility and safety of non-intubated thoracoscopic surgery in spontaneously breathing patients, using regional block analgesia.

## Material and Methods

### Patients and study design

This was a prospective, uncontrolled, open-label study of consecutive patients scheduled for thoracoscopic surgery between April 2013 and June 2015 at the Department of Anesthesiology of Shenzhen People's Hospital (China). The inclusion criteria were: 1) American Society of Anesthesiologists (ASA) class I–II; 2) <70 years of age; 3) body mass index (BMI) <28 kg/m^2^; 4) no history of cardiovascular disease; and 5) no history of bronchial dilatation, pulmonary abscess, tuberculosis, or pulmonary disease that required lung isolation. The exclusion criteria were: 1) cold, fever, or cough; 2) chronic bronchitis, asthma, or any respiratory disorder leading to decreased lung function; 3) history of cranial surgery or brain trauma; 4) history of cognitive or mental disorders; 5) epilepsy or any other nervous system disease; 6) history of medication allergy; 7) thoracic adhesions requiring complex surgery; or 8) large expected amount of blood loss. Those requiring conversion to endotracheal intubation during surgery due to adverse events such as a large amount of blood loss or severe infection during the perioperative period, and those in whom the procedures were not strictly implemented for whatever reason were excluded from the analyses.

The ethics committee of Shenzhen People's Hospital (China) approved the study. Written informed consent was obtained from all participants or their legal guardians.

### Anesthesia and surgery

Anesthesia was carried out by the same anesthesiologist for all patients (a senior clinician with 22 years of experience in anesthesiology who performs anesthesia for thoracic surgery in about 300 patients per year). The patient was placed in the lateral position (pulmonary surgery), supine position (mediastinal surgery), or beach chair position (thoracic sympathicotomy for palmar hyperhidrosis). Scopolamine (0.3 mg) was given by intravenous injection. Electrocardiogram and oxygen saturation (SPO_2_) were strictly monitored throughout the procedure. Radial artery puncture was performed under topical anesthesia to sample arterial blood and allow direct measurement of arterial blood pressure. Dexmedetomidine hydrochloride (1.0 µg · kg^-1^ · h^-1^; lot #16120832; Jiangsu Hengrui Medicine Co. Ltd., China) was given for sedation, using a venous pump. Propofol (lot #1612099, AstraZeneca, UK) and remifentanil (Renfu Pharmaceutical Co. Ltd, China) were administered 10 min later for anesthesia and analgesia using a target-controlled infusion (TCI) pump. The rate of the TCI was adjusted according to the bispectral index (BIS) (monitored throughout surgery), breathing, circulatory parameters, and responses to surgical stimuli, based on the anesthesiologist's experience. When the BIS index reached 50±10, a laryngeal mask airway was used for patients undergoing surgery for mediastinal tumor or palmar hyperhidrosis, while a nasopharyngeal airway was used for those undergoing pulmonary lobectomy or lung wedge resection. The anesthesia machine was used to deliver 50% oxygen at 5 L/min. The end-tidal partial pressure of carbon dioxide (ETCO_2_) was measured using a sampling tube connected to the airway. Prior to surgery, the TCI rate was adjusted to ensure a BIS value of 40–55.

**Figure 1:**
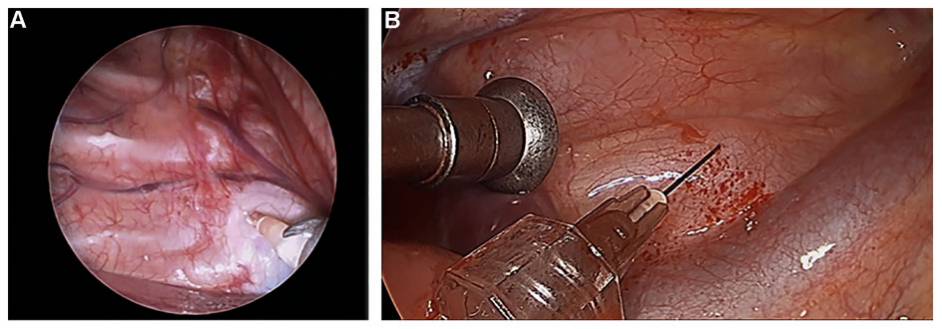
Representative images obtained during surgery showing (**A**) paravertebral block and (**B**) vagus nerve block in the right chest cavity. In all cases, regional block was administered under direct thoracoscopic vision.

Intercostal nerve block was performed using 8 mL of 0.4% ropivacaine (AstraZeneca) for each segment. For patients undergoing pulmonary lobectomy, segmentectomy, or lung wedge resection, paravertebral block was achieved with 0.4% ropivacaine (3 mL for each segment) to the T4–T6 segments. Another round of paravertebral block and vagal block was administered if the surgery lasted >150 min. Intrathoracic vagal block with 3 mL of 2% lidocaine was used to prevent coughing during thoracoscopy (Figure 1).

All operations were carried out by the same surgeon, an associate chief clinician with 20 years of experience in thoracic surgery. Single-lumen thoracoscopy was used for segmentectomy or lung wedge resection. Multi-lumen thoracoscopy was used for lobectomy and mediastinal tumor excision. Bilateral sympathicotomy was performed under thoracoscopy for patients diagnosed with palmar hyperhidrosis.

Intraoperatively, mean arterial pressure (MAP) was maintained at >55 mmHg, and dopamine was administered if necessary. At the end of surgery, the TCI rate of propofol was gradually reduced. The administration of propofol, remifentanil, and dexmedetomidine was stopped within 3 min of skin suture. The laryngeal mask or nasopharyngeal airway was removed after awakening.

### Data collection

The following baseline characteristics were recorded: age, sex, and indication for surgery. MAP, HR, SPO_2_, and ETCO_2_ were recorded at the following time points: before the induction of anesthesia (pre-anesthesia), immediately after placing the airway (airway insertion), immediately after skin incision (skin incision), 10 min after opening the chest cavity (10 min post-chest access), immediately after the end of surgery (post-surgery), and immediately after removal of the airway (airway removal). Blood gas analysis was performed using blood from the radial artery before anesthesia, 30 min after opening the chest cavity, and 5 min after consciousness recovery. These time points were chosen to reflect blood gas composition before, during, and after anesthesia. The duration of surgery was defined as from anesthesia induction to removal of the airway. The Quality of Recovery-15 (QoR-15) scale (14) and the Visual Analog Scale (VAS) (15) were used to evaluate recovery and pain status at 6 and 24 h after surgery. The two scales were administered by the same clinician with 10 years of experience in anesthesiology. The QoR-15 scale uses 15 questions to assess five domains of patient-reported health status (pain, physical comfort, physical independence, psychological support, and emotional state); the score ranges from 0 (very poor recovery) to 150 (excellent recovery) (14). The VAS is an 11-point numeric scale with 0 representing "no pain" and 10 representing "the worst pain imaginable" (15).

### Outcome measures

The primary outcome measures were the changes in MAP, HR, SPO_2_, and ETCO_2_ among the six time points (pre-anesthesia, airway insertion, skin incision, 10 min post-chest access, post-surgery, and airway removal). The secondary outcome measures were the changes in blood gas values among the three time points (pre-anesthesia, 30 min post-chest cavity access, and 5 min after recovery) and the QoR-15 scale and VAS scores at 6 and 24 h after surgery.

### Safety

Any perioperative complications, including anesthesia-related complications, were noted.

### Statistical analysis

Data analysis was performed using SPSS 13.0 (IBM, USA). Continuous data are reported as means±SD; within-group comparisons over time were carried out using repeated measures analysis of variance (ANOVA) with the Bonferroni *post hoc* test. Categorical data are reported as absolute and relative frequencies, and were analyzed using the chi-squared test or Fisher's exact test. Two-sided P-values <0.05 were considered statistically significant.

## Results

### Characteristics of the patients

Fifty-seven consecutive patients were included in the study. Table 1 presents their characteristics. Among them, 30 were male and 27 were female. The patients were 16–63 (mean, 42.3±19.5) years of age and were ASA I or II. Among them, six underwent pulmonary lobectomy for lung cancer, 15 underwent segmentectomy or wedge resection for pulmonary nodules, 13 were treated for a mediastinal tumor, and 23 underwent bilateral sympathicotomy for palmar hyperhidrosis. The duration of surgery was 40–240 (mean, 116.8±38.1) min.

**Table 1:** Characteristics of the patients.

Characteristic	Value (n=57)
Age (years), mean±SD	42.3±19.5
Male sex, n (%)	30 (52.6)
Lobectomy for lung cancer, n (%)	6 (10.5)
Segment or wedge resection for pulmonary nodule, n (%)	15 (26.3)
Resection of mediastinal tumor, n (%)	13 (22.8)
Thoracic sympathicotomy for palmar hyperhidrosis, n (%)	23 (40.4)

SD: standard deviation.

Before surgery, routine blood, electrolyte, hepatic function, and renal function tests showed normal results in all patients. Heart and lung functions were also normal in all patients. One case was excluded from the analysis because of conversion to double-lumen endotracheal intubation and mechanical ventilation due to injury to a pulmonary artery branch that caused a massive hemorrhage; the conversion to general anesthesia, intubation, and one-lung ventilation in this patient was deemed necessary to ensure a clear surgical field and to stop the hemorrhage quickly.

In all 13 patients with mediastinal tumor, pathology revealed a thymoma and lymph node dissection was not performed. The six patients undergoing pulmonary lobectomy were found to have malignant tumors and underwent lymph node dissection under thoracoscopy.

### Hemodynamic parameters

Table 2 presents the hemodynamic parameters over time. Two patients with mediastinal tumors developed intraoperative hypoxemia with SPO_2_ <90%. The MAP before induction of anesthesia and after removal of the laryngeal mask or nasopharyngeal airway was significantly higher than that at the four intraoperative time points (all P<0.05). The MAP at 10 min after opening the chest was considerably lower than that at other time points (all P<0.05). The HR before induction of anesthesia and after removal of the airway was significantly higher than that at the four intervention time points (all P<0.05). SPO_2_ did not differ significantly among time points. The ETCO_2_ after removal of the airway was significantly lower than that measured for the preceding intraoperative time points (all P<0.05). Intraoperatively, 15 patients required dopamine to maintain MAP >55 mmHg.

**Table 2:** Comparison of hemodynamic parameters and end-tidal partial pressure of carbon dioxide among different time points.

Parameter	Pre-anesthesia	Airway insertion	Skin incision	10 min post-chest access	Post-surgery	Airway removal
MAP	87.3±9.1^a^	64.1±8.1^b^	64.6±8.0^b^	59.6±6.4^c^	67.6±8.7^b^	84.8±8.4^a^
HR	80±8^a^	66±7^b^	61±5^b^	61±7^b^	68±8^b^	88±7^a^
SPO_2_	98.2±1.0^a^	98.6±1.0^a^	97.7±1.0^a^	97.1±1.6^a^	97.8±1.5^a^	98.1±1.4^a^
ETCO_2_	-	55.8±8.5^a^	59.9±6.8^a^	60.1±5.7^a^	61.1±6.1^a^	45.0±6.9^b^

Data are reported as means±SD. MAP: mean arterial pressure; HR: heart rate; SPO_2_: partial pressure of oxygen; ETCO_2_: end-tidal partial pressure of carbon dioxide. Within-group comparisons over time were carried out using repeated measures analysis of variance (ANOVA) with the Bonferroni *post hoc* test. Different superscript letters denote significant differences (P<0.05) between time points for a specific parameter.

### Blood gas analysis

The pH, PCO_2_, and lactic acid levels 30 min after chest cavity access were significantly different from those measured before induction of anesthesia and 5 min after recovery of consciousness (all P<0.05), whereas no difference was observed between the pre-anesthesia and 5 min after recovery of consciousness time points (Table 3).

**Table 3:** Comparison of arterial blood gas values between different time points.

Parameter	Pre-anesthesia	30 min post-chest cavity access	5 min after recovery
pH	7.34±0.07^a^	7.18±0.18^b^	7.32±0.08^a^
PCO_2_ (mmHg)	42.3±3.9^a^	64.1±6.2^b^	45.2±5.1^a^
Lactic acid (mmol/L)	0.99±0.15^a^	2.58±1.81^b^	1.28±0.26^a^
Glucose (mmol/L)	5.67±1.66^a^	6.89±2.54^a^	6.51±1.86^a^

Data are reported as means±SD. PCO_2_: partial pressure of carbon dioxide. Within-group comparisons over time were carried out using repeated measures analysis of variance (ANOVA) with the Bonferroni *post hoc* test. Different superscript letters denote significant differences (P<0.05) between time points for a specific parameter.

### QoR-15 and VAS scores after surgery

QoR-15 score at 24 h was significantly lower than that before surgery and at 6 h after surgery (P<0.05). Pain gradually increased after surgery (Table 4).

**Table 4:** Comparison of preoperative and postoperative Quality of Recovery-15 (QoR-15) and Visual Analog Scale (VAS) scores.

Parameter	Preoperative	6 h Postoperative	24 h Postoperative
QoR-15	142.8±3.7^a^	140.1±4.9^a^	132.6±8.1^b^
VAS	0.0±0.0^a^	2.0±0.1^b^	5.0±3.7^c^

Data are reported as means±SD. Within-group comparisons over time were carried out using repeated measures analysis of variance (ANOVA) with the Bonferroni *post hoc* test. Different superscript letters denote significant differences (P<0.05) between time points for a specific parameter.

### Adverse events

All 57 patients remained stable during the perioperative period. No anesthesia-related complications were documented. One patient had pulmonary artery branch hemorrhage during pulmonary lobectomy and the procedure was converted to intubated double lung ventilation; this patient was excluded from the analyses presented above. Another patient undergoing pulmonary lobectomy for >150 min had choking cough during surgery that was treated with paravertebral block using half-dose ropivacaine and vagal nerve block using lidocaine. Three patients undergoing segmentectomy and wedge-shaped resection of the lung, 11 undergoing sympathicotomy for palmar hyperhidrosis, and one undergoing resection of a mediastinal tumor had hypotension (MAP <55 mmHg) during surgery, which was managed intraoperatively by the administration of dopamine.

## Discussion

The present study was designed to evaluate the feasibility and safety of thoracoscopic surgery under non-intubated general anesthesia. An important finding was that paravertebral and thoracic vagal block could be achieved successfully under direct vision through a thoracoscope. Intraoperative and postoperative complications related to non-intubated anesthesia were uncommon and non-serious, with only one patient requiring conversion to intubated anesthesia due to hemorrhage. These results suggest that non-intubated anesthesia for thoracoscopic surgery was feasible and safe.

Only a small number of previous studies explored the use of non-intubated anesthesia for thoracic surgery (summarized in Supplementary Table S1), with most of these focusing on epidural anesthesia. Thoracoscopic surgery under epidural anesthesia has been reported to be safe and effective, with previous studies describing no major perioperative or postoperative complications (2,6,8,9,16–20). Compared with conventional techniques using intubated general anesthesia and one-lung ventilation, non-intubated thoracoscopic surgery with epidural anesthesia has been reported to have advantages that include shorter operation time, better patient satisfaction, less nursing care, shorter postoperative fasting time, shorter duration of antibiotic use, shorter hospital stay, and lower rate of sore throat (2,8,9,17,18). Other clinical investigations demonstrated the feasibility and safety of non-intubated thoracoscopic surgery with paravertebral or intercostal nerve block (3,5,21–23). Most studies of non-intubated regional block and epidural anesthesia found both methods to be broadly comparable with regard to feasibility, safety, and cost (3,5,21), although one study reported advantages of regional block that included faster induction of anesthesia, shorter operation time, shorter duration of chest tube drainage, more stable intraoperative hemodynamics, less blood loss, and shorter hospital stay (22). Previously reported values for the rate of conversion to intubated general anesthesia have ranged from 0–10% (2,3,5,6,8,9,16–24), supporting the value determined in the present study (<2%). In addition, the mean operation time in this study (117 min) was similar to that described in other publications (69–185 min) (6,16,19,22,23). Taken together, these results indicate that non-intubated anesthesia for thoracoscopic surgery is feasible and safe.

Cephalic displacement of the diaphragm during conventional intubated general anesthesia causes a decrease in functional residual capacity, and mechanical ventilation with positive end-expiratory pressure is required to maintain sufficient ventilation and avoid atelectasis of the dependent lung (25). In contrast, non-intubated VATS allows patients to breathe spontaneously, reducing the impact on diaphragmatic function and functional residual capacity of the dependent lung (26,27). In the present study, 55 of the 57 patients maintained SPO_2_ >98% throughout the procedure. The remaining two patients had mediastinal tumors and presented with intraoperative hypoxemia (SPO_2_ <90%) due to surgical injury to the contralateral pleura, leading to an artificial pneumothorax and some collapse of the non-operated lung; their SPO_2_ was successfully restored after repair of the pleural injury and assisted ventilation. The low rate of intraoperative hypoxemia in this study is in agreement with previous investigations (9,21,22). Thus, SPO_2_ can be maintained at a relatively high level in the majority of patients treated with thoracoscopic surgery under non-intubated anesthesia. On the other hand, in this study, all patients presented with carbon dioxide accumulation. ETCO_2_ ranged from 60–70 mmHg, although in one patient ETCO_2_ reached 80 mmHg. Although the occurrence of hypercapnia did not result in any complications and ETCO_2_ recovered to preoperative levels by 5 min after completion of surgery, attention should be paid to this potential issue.

Since hypoxia and hypercapnia remain a risk in spontaneously breathing patients undergoing non-intubated anesthesia, establishing an artificial airway is important to ensure patient safety. We selected a laryngeal mask or nasopharyngeal airway for this purpose. We recommend a laryngeal mask for patients with mediastinal tumors and those with palmar hyperhidrosis, for whom the surgical incision is extremely small and more time is needed to control breathing during the expulsion of residual gas from the thorax. For patients undergoing lobectomy, segmentectomy, or wedge resection of the lung, we suggest that a nasopharyngeal airway is preferable. Further research will be needed to establish the optimal technique.

Many patients in this study showed intraoperative hypotension, especially those with palmar hyperhidrosis undergoing bilateral sympathicotomy. The high incidence of hypotension may be associated with mediastinal swing (8), in part caused by the injection of carbon dioxide to facilitate lung collapse and enlarge the visual field. Other factors possibly contributing to hypotension in these patients included performing surgery in the afternoon, which necessitated a longer fasting time, and positioning during surgery, which would have decreased venous return. We recommend raising the lower extremities during surgery and using vasoactive agents when necessary to maintain blood pressure at an appropriate level. In our experience, a small dose of dopamine (3–5 µg · kg^-1^ · min^-1^) can be administered when necessary to elevate the blood pressure to an acceptable level.

In this study, the mean VAS score was relatively low at 6 h (2.0±0.1) but was increased at 24 h (7.8±0.5), probably due to the wearing off of the regional block. Many patients had intercostal muscle traction during surgery, leading to postoperative intercostal neuralgia. The QoR-15 provides an efficient evaluation of the quality of postoperative recovery (14). In this study, the QoR-15 score did not differ at 6 h after surgery compared with the preoperative levels, however, it was significantly lower at 24 h after surgery, mainly in pain-related items, which is consistent with the changes in VAS score. These results suggest that successful regional block was achieved during thoracic surgery, but that pain after surgery occurred due to weakening of the regional block, intercostal neuralgia, and perhaps other hyperalgesic factors. Attention should be paid to administering sufficient levels of analgesia during the recovery period.

Coughing is usually prevented during intubated general anesthesia because of neuromuscular blockade but can be triggered by excessive stretch of lung parenchyma or hilar manipulation of bronchial structures during non-intubated procedures (1). Unexpected coughing during non-intubated thoracic procedures not only impedes surgical exposure but also potentially causes life-threatening injuries to intra-thoracic structures (9). Therefore, it is advisable to manipulate the lung parenchyma and hilar structures gently during surgery to avoid cough reflex during non-intubated anesthesia, particularly during major and lengthy procedures such as lobectomy or segmentectomy (16). We hypothesize that continuous regional block or self-controlled intravenous analgesia might partially resolve this issue, but this remains to be established.

The present study has limitations. The sample size was small and from a single center. In addition, there was no comparison group. Additional studies, ideally randomized controlled trials, are necessary to determine the efficacy and safety of non-intubated anesthesia for thoracoscopy.

We concluded that paravertebral and thoracic vagal nerve block can be achieved under direct vision through a thoracoscope. Non-intubated anesthesia for thoracoscopic surgery was feasible and safe and reduced the impact of surgery on the patient.

## Supplementary Material

Click here to view [pdf].
